# Supplementary material for: Analysis of PPARγ Signaling Activity in Psoriasis
Source: Int J Mol Sci. 2021 Aug 10;22(16):8603. doi: 10.3390/ijms22168603 (PMC8395241; doi:10.3390/ijms22168603)
Supplement: Supplementary file 1 [file ijms-22-08603-s001.zip › Supplemental materials_Analysis of PPARg signaling activity in psoriasis/Pathway models/Models images and html files/Differentiation of psoriatic T cells/11538.html]

TNFRSF1A


# Protein TNFRSF1A

|  |  |
| --- | --- |
| URN | urn:agi-llid:7132 |
| Total Entities | 0 |
| Connectivity | 4233 |
| Name | TNFRSF1A |
| Description | tumor necrosis factor receptor superfamily, member 1A |
| Notes | This gene encodes a member of the TNF receptor superfamily of proteins. The encoded receptor is found in membrane-bound and soluble forms that interact with membrane-bound and soluble forms, respectively, of its ligand, tumor necrosis factor alpha. Binding of membrane-bound tumor necrosis factor alpha to the membrane-bound receptor induces receptor trimerization and activation, which plays a role in cell survival, apoptosis, and inflammation. Proteolytic processing of the encoded receptor results in release of the soluble form of the receptor, which can interact with free tumor necrosis factor alpha to inhibit inflammation. Mutations in this gene underlie tumor necrosis factor receptor-associated periodic syndrome (TRAPS), characterized by fever, abdominal pain and other features. Mutations in this gene may also be associated with multiple sclerosis in human patients. [provided by RefSeq, Sep 2016] |
| Primary Cell Localization | Plasma membrane |
| Class | Receptor |

---

|  |  |
| --- | --- |
| Pathway | Alzheimer's Disease |
|  | Osteoarthritis |
|  | Skin Aging |
|  | MTHFR Activity Regulation |
|  | mTOR Signaling Hyperfunction |
|  | TLR4 Signaling in Leukocytes |
|  | Peripheral T-Cell Tolerance |
|  | T-Cell Cytotoxic Mediated Cell Death |
|  | Necrosis |
|  | Leukotriene Effect on Vascular Endothelial Cell Response |
|  | Eosinophil Survival by Cytokine Signaling |
|  | Macrophage M1 Lineage |
|  | Neutrophil Recruitment and Priming |
|  | Negative Acute Phase Proteins Synthesis |
|  | Positive Acute Phase Proteins Synthesis |
|  | Vascular Endothelial Cell Activation by Cytokines |
|  | Adipokines Production by Adipocyte |
|  | Lipolysis Regulation in Adipocyte |
|  | Apoptosis |
|  | Proteins Involved in Atherosclerosis |
|  | Dilated Cardiomyopathy |
|  | Cardiomyocyte Hypertrophy |
|  | Hypertrophic Cardiomyopathy |
|  | Myocardial Infarction |
|  | Proteins Involved in Myocarditis |
|  | Apoptosis of Myocyte |
|  | SOCS in Negative Feedback Regulation in Myocarditis |
|  | Inflammatory Reaction in Acne Vulgaris |
|  | Hair Follicle Keratinocyte Apoptosis |
|  | IFN-gamma/TNF-alpha Mediated Cell Proliferation in Psoriasis |
|  | Th0-Cell Aberrant Activation in Systemic Lupus Erythematosus |
|  | Oxidative Stress in Vitiligo |
|  | Vitiligo |
|  | Toll-like Receptors in beta-Cell |
|  | Podocyte Dysfunction in Diabetic Nephropathy |
|  | Adipokines and Cytokines in Insulin Resistance in Skeletal Muscles |
|  | Proteins Involved in Insulin Resistance |
|  | Adipose Tissue Inflammation Provokes Insulin Resistance |
|  | Adiponectin Synthesis Declines in Insulin Resistance |
|  | Proteins Involved in non-Alcoholic Fatty Liver Disease |
|  | Oxidation of Fatty Acid in non-Alcoholic Fatty Liver Disease |
|  | beta-Cell Destruction through Cytokines in Diabetes Mellitus |
|  | beta-Cell Destruction in Diabetes Mellitus Type 1 |
|  | Adipokines Production by Adipocyte Impaired in Obesity |
|  | Proteins Involved in Obesity |
|  | Crohn's Disease |
|  | Macrophage Dysfunction |
|  | Proteins Involved in B-Cell Chronic Lymphocytic Leukemia |
|  | Mantle Cell Lymphoma |
|  | mTOR/NF-kB/BCR Signaling Disregulation in Mantle Cell Lymphoma |
|  | Splenic Marginal Zone Lymphoma |
|  | TNFR/NF-kB Classical Pathway in Multiple Myeloma Plasma Cells |
|  | Multiple Myeloma |
|  | TNFR/NF-kB Alternative Pathway in Multiple Myeloma Plasma Cells |
|  | Goblet-Cell Related Mucus Secretion in Asthma |
|  | Proteins Involved in Cystic Fibrosis |
|  | Airway Epithelial Cell Dysfunction |
|  | Neutrophil Recruitment in Sinovium in Gout |
|  | TNF and IL1B Induce Metalloproteinase Synthesis in Osteoarthritis |
|  | Apoptosis of Chondrocyte |
|  | Dendritic Cell Dysfunction in Psoriatic Arthritis |
|  | Synovial Fibroblast Activation in Psoriatic Arthritis |
|  | Keratinocyte Activation in Psoriatic Arthritis |
|  | Synovial Fibroblast Activation by Citokines in Rheumatoid Arthritis |
|  | Apoptosis Block in Synovial Fibroblast |
|  | Skin Fibrosis |
|  | Dentin and Enamel Dysruprion in Bloch-Sulzberger Syndrome |
|  | Apoptosis Block in Infected Cells |
|  | Diabetes Induced Periodontitis |
|  | IL1 Receptor Antagonist (IL1RN) Deficiency (DIRA) |
|  | TNF Receptor Associated Periodic Syndrome |
|  | Osteoclast Activation in Postmenopause |
|  | Neurofibrillary Tangle Formation |
|  | Apoptosis and Immediate Early Gene Activation in Epileptiform Disorders |
|  | Glioblastoma, Mesenchymal Subtype |
|  | Crystallin alpha B (CRYAB) Suppresses Multiple Sclerosis |
|  | Proteins Involved in Endometriosis |
|  | Subepithelial Fibroblasts in Middle Ear Cholesteatoma |
|  | HPV Infection and Cancer |
|  | HPV E6 and E7 Induced Disruption of TNFR/NF-kB Signaling in Keratinocytes |
|  | OPTN Effects on NF-kB Signaling in Glaucoma |
|  | Glutamate Mediated Excitotoxicity in Glaucoma |
|  | Proteins Involved in Otitis Media |
|  | Cytokines Trigger Otitis Media |
|  | Alveolar Epithelial Cell Apoptosis in COPD |
|  | Alveolar Macrophages Dysfunction in COPD |
|  | Mucin Hyperproduction in Goblet and Mucous Cells |
|  | Proteins Involved in Glomerulonephritis |
|  | Podocyte Dysfunction in Glomerulonephritis |
|  | Interstitial Fibroblasts in Pyelonephritis |
|  | Apoptosis of Renal Tubule Epithelial Cell in Pyelonephritis |
|  | Dopamine/Gs Expression Targets |
|  | Dopamine/Gi Expression Targets |
|  | Elevated Receptors -> Expression Targets in Aorta |
|  | CXCL12 Expression Targets |
|  | FSHR Expression Targets |
|  | NFKB Canonical Signaling Activation in Cancer |
|  | NFKB Non-Canonical Signaling Activation in Cancer |
|  | STAT3 and NFkB Activate Inflammation-Induced Tumorigenesis |
|  | NFKB Signaling Activation by Blocking of Tumor Suppressors |
|  | Apoptosis Block by Death Receptor Signaling in Cancer |
|  | Apoptosis Evasion in Cancer: Overview |
|  | Cancer Cells Inhibit Adipocyte Differentiation |
|  | Skeletal Muscle Wasting in Cancer Cachexia |
|  | IDO1 in Cancer Immune Escape |
|  | Effector T-cell Inactivation in Cancer Immune Escape |
|  | IFNG/IFNR Expression Targets |
|  | IL2 Expression Targets |
|  | Interleukin Receptors -> Expression Targets in Lymphoid System and Blood |
|  | CD40 -> Expression Targets in Thymus |
|  | CD40 -> Expression Targets in Lymph Node |
|  | CSF1 -> STAT Expression Targets |
|  | CSF1 -> AP-1/CREB/CREBBP/MYC Expression Targets |
|  | TNF-alpha/TNFRSF1A Signaling |
|  | Ras-GAP Regulation Signaling |
|  | NF-kB non-Canonical Signaling |
|  | NF-kB Canonical Signaling |
|  | TGFB1-ACVRL1 Expression Targets |
|  | TNF -> NF-kB Expression Targets |
|  | LTA Expression Targets |
|  | TNF -> AP-1 Expression Targets |
|  | TNF -> ELK-SRF Expression Targets |
|  | TNF -> CREB Expression Targets |
|  | TNF -> STAT Expression Targets |
|  | TNF -> TP53/ATF Expression Targets |
|  | TNFRSF1A -> STAT Signaling |
|  | TNFR -> NF-kB Signaling |
|  | TNFRSF1A -> AP-1/ATF/TP53 Signaling |
|  | TNFRSF1A -> CREB/ELK-SRF Signaling |
|  | IL1B -> NO Expression Targets |
|  | TLR4 -> AP-1/EGR1/HIF1A Expression Targets |
|  | IL1B Expression Targets |
|  | IL1A Expression Targets |
|  | Model of PPARG signaling in psoriasis |
|  | 1\_Inflammation in keratinocytes in the pathology of acne |
|  | 1\_Differentiation of psoriatic T cells |
|  | 1\_TNF and IL1B provoke ECM degradation in osteoarthritis |
|  | 1\_Osteoclast-mediated bone resorption in postmenopausal women |
|  | 2\_Chondrocyte death in osteoarthritis |
|  | 3\_The role of interstitial fibroblasts in pyelonephritis |
|  | 2\_Podocyte dysfunction in glomerulonephritis |
|  | 2\_Pathogens stimulate mucins expression in the middle ear |
|  | 2\_Cardiomyocyte hypertrophy |
|  | 2\_Cigarette smoke and inflammation cause alveolar epithelial cell death in COPD |
|  | 3\_Role of alveolar macrophages in pulmonary tissue destruction in COPD |
|  | 3\_2\_Regulation of airway surface liquid (ASL) thickness |
|  | 3\_1\_Mucus accumulation in asthma |
|  | 4\_Mucin hyperproduction in goblet and mucous cells in COPD |
|  | 4\_1\_Polymorphisms associated with inflammatory bowel diseases |
|  | 6\_p12I Viral protein promotes T-cell activation in HTLV-1 infection |
|  | 5\_Human papilloma virus E6 and E7 proteins promote epithelial cell proliferation |
|  | 2\_HIV - induced depletion of CD4+ T-cells |
|  | 4\_Inflammatory mediators inhibit GATA signaling in erythropoiesis |
|  | 4\_2\_Insulin resistance: inflammation-related insulin resistance |
|  | 2\_Ion channel CFTR failure in cystic fibrosis airway epithelium (class III-IV mutations) |
|  | 1\_Antigen-presenting cells promote islet dysfunction and immune system activation in T1D |
|  | 3\_Islet beta-cell destruction in T1D |
|  | 2\_Neuron and Schwann cell death |
|  | drug-target molsynthesis |
|  | Differentiation of psoriatic T cells |

---

|  |  |
| --- | --- |
| Group | Genes Associated with Systemic Lupus Erythematosus |

---

|  |  |
| --- | --- |
| MedScan ID | 7132 |

---

|  |  |
| --- | --- |
| LocusLink ID | 7132 |
|  | 21937 |
|  | 25625 |

---

|  |  |
| --- | --- |
| Alias | tumor necrosis factor TNF receptors |
|  | Tnfrh3 |
|  | TNF-alphaR1 |
|  | TBPI |
|  | TNFRp55 |
|  | tumor necrosis factor receptor 1A isoform beta |
|  | tumor necrosis factor receptor 1 |
|  | TRAPS gene |
|  | CD120a antigen |
|  | TNF-R-I |
|  | LOC113298 |
|  | tumor necrosis factor TNF receptor |
|  | TNF receptor alpha chain |
|  | TNF receptor 1 |
|  | tumor necrosis factor receptor type I |
|  | MGC19588 |
|  | LOC116290 |
|  | TR1A |
|  | GNB:339760 |
|  | TR1A\_HUMAN |
|  | TNFRI |
|  | TNF-R55 |
|  | TNFAR |
|  | TNFR60 |
|  | TNFRSF1A |
|  | TNFR1-d2 |
|  | CD120A |
|  | MGC105478 |
|  | TNFR55 |
|  | OTTMUSP00000028139 |
|  | TNF receptor 1A |
|  | TNF receptor superfamily member 1A |
|  | Familial periodic fever (Hibernian fever) gene |
|  | tumor necrosis factor-alpha receptor |
|  | TNFR1 |
|  | TNF-R |
|  | TNF receptors |
|  | tumor necrosis factor receptor superfamily, member 1A |
|  | TNF-RI |
|  | TNFR I |
|  | TNF-R1 |
|  | TNF receptor homolog 3 |
|  | Tumor necrosis factor binding protein 1 |
|  | Tumor necrosis factor binding protein I |
|  | tumor necrosis factor receptor 1 (55kD) |
|  | tumor necrosis factor, alpha receptors |
|  | TNF receptor |
|  | p55-R |
|  | TNF receptor homolog III |
|  | FPF |
|  | p55 |
|  | p60 |
|  | TBP1 |
|  | TNFR-I |
|  | tumor necrosis factor receptor type 1 |
|  | tumor necrosis factor receptor superfamily member 1A |
|  | Tnfr-2 |
|  | TNFalpha-R1 |
|  | TNF-alpha-R1 |
|  | TNFR-1 |
|  | tumour necrosis factor receptor 1 (55kD) |
|  | tumour necrosis factor receptor 1A isoform beta |
|  | tumour necrosis factor receptor superfamily, member 1A |
|  | tumour necrosis factor binding protein 1 |
|  | tumour necrosis factor, alpha receptors |
|  | tumour necrosis factor receptor type I |
|  | tumour necrosis factor TNF receptors |
|  | tumour necrosis factor receptor 1 |
|  | tumour necrosis factor binding protein I |
|  | tumour necrosis factor TNF receptor |
|  | tumour necrosis factor-alpha receptor |
|  | MS5 |
|  | TRAPS |
|  | Tumor necrosis factor receptor 1 precursor |
|  | TNF-R1s |
|  | TNFR60s |
|  | TNFR55s |
|  | Tnfrh3s |

---

|  |  |
| --- | --- |
| GO ID | 0043120 |
|  | 0005031 |
|  | 0007249 |
|  | 0003176 |
|  | 0071260 |
|  | 0019221 |
|  | 0071550 |
|  | 0042742 |
|  | 0008625 |
|  | 0006954 |
|  | 0008630 |
|  | 0010614 |
|  | 0003332 |
|  | 0050728 |
|  | 0043123 |
|  | 1902339 |
|  | 2000304 |
|  | 0050729 |
|  | 0045944 |
|  | 0042531 |
|  | 0006693 |
|  | 0072659 |
|  | 0003177 |
|  | 1903140 |
|  | 0010803 |
|  | 0033209 |
|  | 0016032 |
|  | 0000139 |
|  | 0009986 |
|  | 0005576 |
|  | 0005615 |
|  | 0005887 |
|  | 0016020 |
|  | 0045121 |
|  | 0005739 |
|  | 0005886 |
|  | 0043235 |
|  | 0002947 |
|  | 0002020 |
|  | 0044877 |
|  | 0006915 |
|  | 0007166 |
|  | 0006952 |
|  | 0043066 |
|  | 0010629 |
|  | 0032715 |
|  | 0045766 |
|  | 0010628 |
|  | 0043525 |
|  | 0032760 |
|  | 0051291 |
|  | 0042981 |
|  | 0007165 |
|  | 0005794 |
|  | 0030424 |
|  | 0005829 |
|  | 0016021 |
|  | 0005634 |
|  | 0032991 |
|  | 0045202 |
|  | 0071392 |
|  | 0043279 |
|  | 0043200 |
|  | 0045471 |
|  | 0001666 |
|  | 0032496 |
|  | 0009611 |
|  | 0090002 |
|  | 0006955 |
|  | 0042511 |
|  | 0042127 |
|  | 0032403 |
|  | 0007275 |
|  | 0033160 |
|  | 0043234 |
|  | 0033013 |
|  | 0097190 |
|  | 0012501 |
|  | 0005622 |
|  | 0016101 |
|  | 0009812 |
|  | 0019048 |
|  | 0008624 |
|  | 0004872 |
|  | 0044419 |
|  | 0005515 |

---

|  |  |
| --- | --- |
| KEGG ID | hsa:7132 |
|  | mmu:21937 |
|  | rno:25625 |

---

|  |  |
| --- | --- |
| Organism | Homo sapiens {Organism urn:agi-taxid:9606} |
|  | Mus musculus {Organism urn:agi-taxid:10090} |
|  | Rattus norvegicus {Organism urn:agi-taxid:10116} |
|  | Homo sapiens |
|  | Mus musculus |
|  | Rattus norvegicus |

---

|  |  |
| --- | --- |
| Mouse chromosome position | 6 59.32 cM |
|  | 6 60.55 cM |

---

|  |  |
| --- | --- |
| OMIM ID | 191190 |
|  | 614810 |
|  | 142680 |

---

|  |  |
| --- | --- |
| Rat chromosome position | 4q42 |

---

|  |  |
| --- | --- |
| Hugo ID | 11916 |
|  | HGNC:11916 |

---

|  |  |
| --- | --- |
| Human chromosome position | 12p13.31 |
|  | 12p13.2 |

---

|  |  |
| --- | --- |
| Swiss-Prot Accession | P19438 |
|  | J9PH39 |
|  | P19438.1 |
|  | P25118 |
|  | Q3U479 |
|  | P25118.1 |
|  | P22934 |
|  | P22934.2 |
|  | A8K4X3 |
|  | B2RDE4 |
|  | B3KPQ1 |
|  | B4DQB7 |
|  | B4E309 |
|  | B5M0B5 |
|  | D3DUR1 |
|  | Q9UCA4 |
|  | Q5U1X6 |
|  | Q91V30 |
|  | Q91Y93 |
|  | Q6Y4Q7 |
|  | Q3TVS9 |
|  | Q4VAE0 |
|  | Q6QHF0 |
|  | Q6QHF1 |

---

|  |  |
| --- | --- |
| PIR ID | A38208 |
|  | A38634 |
|  | B36555 |

---

|  |  |
| --- | --- |
| GenBank ID | NC\_000012 |
|  | NR\_144351 |
|  | NM\_001065 |
|  | NP\_001056 |
|  | NM\_001346091 |
|  | NP\_001333020 |
|  | NM\_001346092 |
|  | NP\_001333021 |
|  | NG\_007506 |
|  | AC006057 |
|  | AF047488 |
|  | AH003016 |
|  | AAA61201 |
|  | AY131997 |
|  | AAM77802 |
|  | CH471116 |
|  | EAW88805 |
|  | EAW88806 |
|  | CS068582 |
|  | CAI84622 |
|  | JA362584 |
|  | CCA94574 |
|  | KF455591 |
|  | X69810 |
|  | A21522 |
|  | CAA01558 |
|  | AK056611 |
|  | BAG51763 |
|  | AK130807 |
|  | AK291088 |
|  | BAF83777 |
|  | AK298729 |
|  | BAG60879 |
|  | AK304517 |
|  | BAG65321 |
|  | AK308061 |
|  | AK315509 |
|  | BAG37891 |
|  | AY182755 |
|  | AAO23979 |
|  | BC010140 |
|  | AAH10140 |
|  | BF528949 |
|  | DA010826 |
|  | DA664652 |
|  | EU927389 |
|  | ACH57451 |
|  | JN172914 |
|  | AEO89726 |
|  | M33294 |
|  | AAA03210 |
|  | M58286 |
|  | AAA36753 |
|  | M60275 |
|  | AAA36756 |
|  | M63121 |
|  | AAA36754 |
|  | P19438 |
|  | NC\_000072 |
|  | NM\_011609 |
|  | NP\_035739 |
|  | AC140324 |
|  | AH002101 |
|  | AAA40465 |
|  | CH466523 |
|  | EDK99828 |
|  | L04800 |
|  | S52703 |
|  | AK154388 |
|  | BAE32554 |
|  | AK159989 |
|  | BAE35539 |
|  | AK178992 |
|  | AK181159 |
|  | AK181531 |
|  | AK190321 |
|  | AK206708 |
|  | AK206824 |
|  | AK207044 |
|  | AK211886 |
|  | AY541589 |
|  | AAS48428 |
|  | AY541590 |
|  | AAS48429 |
|  | BC004599 |
|  | AAH04599 |
|  | BC052675 |
|  | AAH52675 |
|  | BC096429 |
|  | AAH96429 |
|  | CK624043 |
|  | L26349 |
|  | AAA59361 |
|  | M59377 |
|  | AAA40464 |
|  | M60468 |
|  | AAA39751 |
|  | X57796 |
|  | CAA40936 |
|  | X59238 |
|  | CAA41922 |
|  | P25118 |
|  | NC\_005103 |
|  | XM\_006237334 |
|  | XP\_006237396 |
|  | NM\_013091 |
|  | NP\_037223 |
|  | AC\_000072 |
|  | AABR07062033 |
|  | AAHX01032867 |
|  | CH473964 |
|  | EDM01847 |
|  | AF329976 |
|  | AAK53562 |
|  | AF329977 |
|  | AAK53563 |
|  | AF329978 |
|  | AAK53564 |
|  | AF329979 |
|  | AAK53565 |
|  | AF329980 |
|  | AAK53566 |
|  | AF329981 |
|  | AAK53567 |
|  | BC086413 |
|  | AAH86413 |
|  | M63122 |
|  | AAA42256 |
|  | P22934 |
|  | NC\_018923 |
|  | AMYH02025949 |
|  | M75864 |
|  | M75865 |
|  | M75866 |
|  | EU176385 |
|  | ABW03836 |
|  | EU176726 |
|  | ABW03527 |
|  | AC\_000028 |
|  | AAHY01057259 |
|  | AAHY01057260 |
|  | M76655 |
|  | M76656 |
|  | M88067 |
|  | AC\_000144 |
|  | ABBA01017638 |
|  | XM\_005253758 |
|  | XP\_005253815 |
|  | XM\_005253759 |
|  | XP\_005253816 |
|  | AABR06033524 |
|  | NT\_009759 |
|  | NW\_925295 |
|  | NW\_001838050 |
|  | AC\_000055 |
|  | CR593985 |
|  | CR596841 |
|  | CR597821 |
|  | CR606215 |
|  | CR622520 |
|  | Q6Y4Q7 |
|  | NT\_039353 |
|  | NW\_001030811 |
|  | Q3TVS9 |
|  | Q3U479 |
|  | Q4VAE0 |
|  | Q6QHF0 |
|  | Q6QHF1 |
|  | NW\_001084832 |
|  | NW\_047696 |

---

|  |  |
| --- | --- |
| Swiss-Prot ID | TNR1A\_HUMAN |
|  | TNR1A\_MOUSE |
|  | TNR1A\_RAT |
|  | Q3U479\_MOUSE |

---

|  |  |
| --- | --- |
| Cell Localization | Cell membrane |
|  | Golgi apparatus membrane |
|  | Secreted |
|  | Plasma membrane |
|  | Membrane |

---

|  |  |
| --- | --- |
| Ensembl ID | ENSG00000067182 |
|  | ENSP00000162749.2 |
|  | ENST00000162749.6 |
|  | ENST00000534885.5 |
|  | ENSMUSG00000030341 |
|  | ENSMUSP00000032491.8 |
|  | ENSMUST00000032491.14 |
|  | ENSRNOG00000031312 |
|  | ENSRNOP00000042371.2 |
|  | ENSRNOT00000048529.4 |
|  | ENSP00000162749 |
|  | ENST00000162749 |
|  | ENSMUSP00000032491 |
|  | ENSMUST00000032491 |
|  | ENSRNOP00000042371 |
|  | ENSRNOT00000048529 |

---

|  |  |
| --- | --- |
| MGI ID | MGI:1314884 |
|  | 1314884 |

---

|  |  |
| --- | --- |
| RGD ID | 621237 |

---

|  |  |
| --- | --- |
| Unigene ID | Hs.279594 |
|  | Hs.713833 |
|  | Mm.1258 |
|  | Mm.474976 |
|  | Rn.11119 |

---

|  |  |
| --- | --- |
| Homologene ID | 828 |

---

|  |  |
| --- | --- |
| Shape | Stick-vertex |

---

|  |  |
| --- | --- |
| IPI ID | IPI00212001 |
|  | IPI00018880 |
|  | IPI00795875 |
|  | IPI00929211 |
|  | IPI00317228 |
|  | IPI00796532 |
|  | IPI00794282 |
|  | IPI00607905 |
|  | IPI00856824 |
|  | IPI00886384 |
|  | IPI00857089 |
|  | IPI00568944 |

---
